# Comparative Metabolome Profile between Tobacco and Soybean Grown under Water-Stressed Conditions

**DOI:** 10.1155/2017/3065251

**Published:** 2017-01-03

**Authors:** Roel C. Rabara, Prateek Tripathi, Paul J. Rushton

**Affiliations:** ^1^Texas A&M AgriLife Research and Extension Center, Dallas, TX 75252, USA; ^2^The Scripps Research Institute, La Jolla, CA 92037, USA

## Abstract

Understanding how plants respond to water deficit is important in order to develop crops tolerant to drought. In this study, we compare two large metabolomics datasets where we employed a nontargeted metabolomics approach to elucidate metabolic pathways perturbed by progressive dehydration in tobacco and soybean plants. The two datasets were created using the same strategy to create water deficit conditions and an identical metabolomics pipeline. Comparisons between the two datasets therefore reveal common responses between the two species, responses specific to one of the species, responses that occur in both root and leaf tissues, and responses that are specific to one tissue. Stomatal closure is the immediate response of the plant and this did not coincide with accumulation of abscisic acid. A total of 116 and 140 metabolites were observed in tobacco leaves and roots, respectively, while 241 and 207 were observed in soybean leaves and roots, respectively. Accumulation of metabolites is significantly correlated with the extent of dehydration in both species. Among the metabolites that show increases that are restricted to just one plant, 4-hydroxy-2-oxoglutaric acid (KHG) in tobacco roots and coumestrol in soybean roots show the highest tissue-specific accumulation. The comparisons of these two large nontargeted metabolomics datasets provide novel information and suggest that KHG will be a useful marker for drought stress for some members of Solanaceae and coumestrol for some legume species.

## 1. Introduction

Producing food is a water-intensive process and agriculture is responsible for consuming about 70% of available fresh water [1]. Hence, it is understandable that limited water availability will be detrimental to food production. Drought is a serious threat to food production and is a global problem which affects 64% of global land area [2, 3]. In the US in 2012, 57% of the total cropland was under severe drought, resulting in the decrease of crop production estimates [4]. Drought frequency and intensity are expected to increase due to looming threat of climate change [5] which is why considerable research has been allotted in order to understand how plants respond to this important stress. One approach to elucidate plants response to water stress is through metabolomics. Metabolomics is a powerful tool to gain insights into how plant metabolic processes are regulated under stressful growing conditions [6]. Metabolites are abundant in plants and in the plant kingdom, and the total metabolites are estimated to range between 200,000 and 1 million. Model plant like* Arabidopsis thaliana* is estimated to produce ~5000 metabolites during its life cycle [6–8]. The metabolome of the plant is the link between genotype and phenotype [9] and is considered the ultimate phenotype of cells deduced by the perturbation of gene expression in response to the environment [8]. It can influence both the gene expression and the protein function of the plant which make metabolomics a central component in elucidating cellular systems and decoding gene functions [8, 10, 11]. Metabolome profiling is an attractive tool for phenotyping plants perturbed by environmental changes because it can detect several molecules from biosynthetically unrelated pathways [12]. Traditionally, metabolic analyses were targeted and were focused on single or specific class of metabolites which could lead to somewhat biased and preordained findings [13, 14]. With global metabolome profiling, it can provide less biased view of the metabolic phenotypes and can lead to the discovery of novel metabolic phenotypes that are missed by traditional targeted analyses [9, 13]. Perturbation in plant metabolism occurs when the plant is subjected to stress and, by dissecting the metabolic pathways involved, would provide an overview on what and how metabolic pathways are being regulated during stress. Several studies reported various mechanisms that plants utilize to cope with water deficits and one of those mechanisms is through osmotic adjustments [13]. Plants actively accumulate compatible solutes during water loss to stabilize proteins and cellular structures and/or maintain positive turgor in the cells [14]. The reduction in osmotic potential can be attributed to accumulation of compounds such as amino acids and polyols and thus enable the plants to maintain turgor and growth under these unfavorable growth conditions [13]. One of the amino acids that accumulate in plants under several types of stresses is proline [15]. Potential functions of proline in stress resistance include osmotic adjustment, protection of cellular structure during dehydration, redox buffering, storage and transfer of reductant, a signaling molecule, and scavenger of reactive oxygen species (ROS) [15].

In this study, we utilize metabolome profiling to provide a global perspective of metabolites perturbation in two species of plants subjected to progressive dehydration referred to hereon as water deficit. This will provide us with comparative information of potential novel candidates in two economically important crop species. Further work on those candidates will provide a better understanding of the role and regulation of metabolites during water deficit.

## 2. Materials and Methods

### 2.1. Plant Materials, Growth Conditions, and Water Deficit Treatments

Tobacco plants were grown and subjected to water stress as outlined by Rabara et al. [16]. Surface-sterilized seeds of tobacco cv. “Burley 21” (donated from the University of Virginia) were germinated on half-strength Murashige and Skoog (MS) (Caisson, USA) agar plates. A week after germination, seedlings were transferred to a minihydroponics setup in sterile MK-5 (Caisson, USA) polycarbonate vessels (6 × 6 × 9.5 cm) filled with half-strength MS liquid medium. Plants were supported by plastic platform with holes to ensure that only the roots are submerged in the media. The plants were grown in the growth room set at 25°C for 2 weeks. They were then subjected to dehydration in a growth room for 20, 40, 60, 120, and 240 minutes by removing them from the liquid media using the support platform. Each time point consisted of three replicates with 20 plants per replicate to ensure that enough tissues were harvested. Roots and leaves were harvested and immediately frozen in liquid nitrogen.

Soybean plants were grown and subjected to water stress as outlined by Tripathi et al. [17]. Soybean cv. “Williams 82” seeds were sown in a pot with vermiculite and covered with plastic wrap until germination. Seedlings were transferred in a plastic container filled with growth media. The plants were grown hydroponically using half-strength Hoagland's solution, maintained at pH 5.8 in a growth chamber set at 25°C. After growing them for 30 days, plants were subjected to water stress by removing them from the media into empty boxes without touching them. Leaves and roots samples were harvested after 30, 60, 120, 180, and 300 min dehydration and immediately frozen in liquid nitrogen and stored at −80°C until further processing. Nine plants were utilized for each time point including unstressed plants (three replicates per time point and three plants per replicate). To check whether plants were still viable at the last time point, sample plants were rehydrated in their respective media for recovery. The plants were considered to have recovered when they recovered from wilting.

### 2.2. Measurement of Plant Physiological Responses

Samples from both leaves and roots were selected for the measurement of osmotic potential. The tissue samples were placed in 1.5 mL centrifuge tubes with a polypropylene frit at the bottom of the tube. The tubes were flash frozen in liquid nitrogen. The samples were then thawed and later centrifuged for 5 min at 4,500 rpm to remove the cell sap. After centrifugation, a 10 *μ*L cell sap sample was withdrawn and placed into a Vapro 5520 osmometer (Wescor, USA) to measure osmotic potential. Measurement of leaf stomatal conductance was performed for each time point (*n* = 6) using SC-1 leaf porometer (Decagon, USA).

### 2.3. Metabolomics and Statistical Analyses

Harvested samples from both plant species were ground to powder and 100 mg of the powdered samples was sent to Metabolon, Inc. (USA), for metabolite identification and analysis using their analysis pipeline. Combinations of three independent platforms were utilized for the global unbiased metabolic profiling as described by Oliver et al. [18]. Compounds were identified by comparison to library entries of purified standards or recurrent unknown entities, a total of over one thousand compounds. Following log transformation and imputation with minimum observed values for each compound, Welch's two-sample* t*-test was used to identify metabolites that differed significantly between experimental groups. Metabolites that achieved statistical significance (*p* ≤ 0.05), as well as those approaching significance (0.05 < *p* < 0.1), were highlighted in the dataset. An estimate of the false discovery rate (*q*-value) was also calculated to take into account the multiple comparisons that normally occur in metabolomic-based studies. The *q*-value describes the false discovery rate; a low *q*-value (*q* < 0.10) is an indication of high confidence in a result.

Comparison between treatment and control was done using Welch's two-sample* t*-test in order to identify significantly different metabolites between the control and water-stressed plants. Correlation analyses between all metabolite pairs were done using Pearson's product-moment correlation (Pearson's *r*).

## 3. Results and Discussion

### 3.1. Physiological Response to Water Stress

Tobacco and soybean plants were grown in hydroponic conditions and subjected to progressive dehydration in order to gain insights into the responses to water stress at the metabolic level. We utilized hydroponics system as our method of choice to infer plants response to water stress because we would like to impose only a single stress in the plant compared to the combinatorial stress (heat and water stresses) observed under field-grown plants. The hydroponic system had been widely used to assess the transcriptional profile of plants under water stress [19–21].

In our study, plants exhibited wilting after 20 and 30 min of dehydration in tobacco and soybean, respectively. To check whether the plants were still viable after prolonged water deficit, plants dehydrated for 240 min (tobacco) and 300 min (soybean) were replaced in the media and observed for recovery. After 24 h, all sample plants from both plant systems were able to recover from wilting.

**Figure 1:**
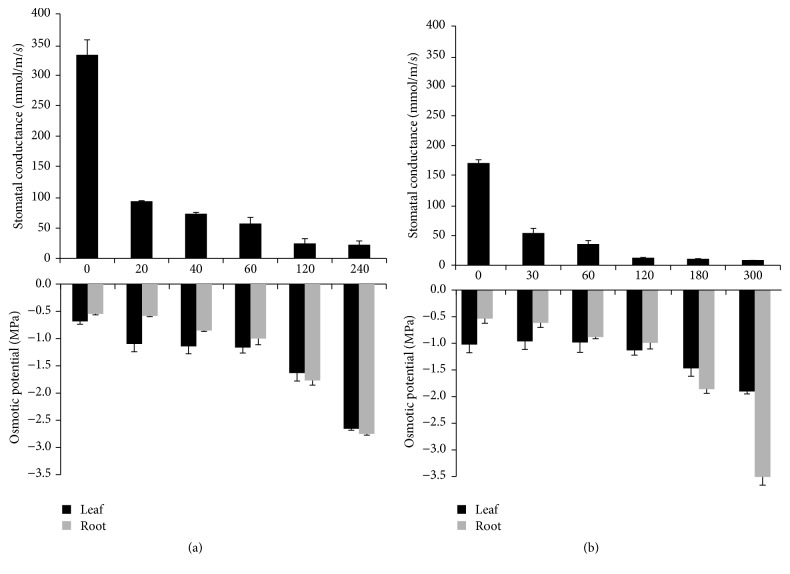
Osmotic potential (MPa) and stomatal conductance (mmol/m/s) of tobacco (a) and soybean (b) at different dehydration time points (min). Horizontal axes are the dehydration time points (min) for each plant species. Data are mean and standard error of three biological replicates.

Tobacco and soybean showed similar physiological responses when subjected to water stress conditions (Figure 1). Both species showed a rapid response to the water stress through closure of stomata as indicated by the measured stomatal conductance. The stomatal conductance dropped significantly after 20 min (tobacco) and 30 min (soybean) of dehydration. Stomatal closure is triggered as an initial response as a means of defense to minimize water loss as transpiration accounts for >90% of water loss in plants [22]. Rapid closure of stomata during water stress is a universal response in plants. Stomatal conductance is an established stress response in plants which has been used as one criterion for selection of drought tolerance and avoidance in* Impatiens capensis* [23] and a reliable tool in screening osmotic stress tolerance in durum wheat [24]. It is also one of the factors that are associated with drought tolerance in grapevines [25]. Reduction of stomatal density has been shown to enhance drought tolerance in* Arabidopsis* by lowering stomatal conductance and transpiration rate [26]. Drought imposition in soybean resulted in a 22% reduction in stomatal density compared to well-watered soybean plants [17].

Similarly, the osmotic potential (OP) in both tissues in both crops showed an almost identical profile. Leaves tend to have low OP values compared to roots in the two species during the early stage of stress (Figure 1). This trend changed during the latter part of the stress wherein roots have lower OP compared to leaves. In tobacco, a rapid decrease (62%) in OP to −1.1 MPa was observed in the leaves but with a little decrease in roots after 20 min dehydration. Further dehydration for 240 min resulted in a 296% decrease in osmotic potential to −2.7 MPa relative to the control. On the other hand, OP of soybean leaves did not show a change until the plants were stressed for 120 min. Soybean OP in both tissues after 300 min of water stress were significantly different with roots reaching −3.5 MPa. In contrast, tobacco leaves and roots have almost similar OP values during the late stage of stress. A difference in osmotic potential between the two tissues in both crops is consistent with the findings of Silveira et al. [27] that a differential response occurred in leaves and roots during osmotic stress. This differential response between the two tissues could be due to their differences in accumulation of osmolytes during water stress but could also be a function of the method of water stress applied.

### 3.2. Effect of Water Stress on the Profile of Metabolites

**Figure 2:**
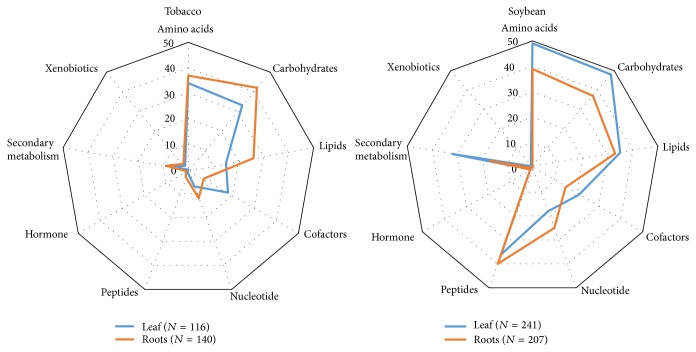
Composition of metabolites detected in leaves and roots of tobacco and soybean plants subjected to time-course based dehydration.

Metabolome profiling was performed in order to gain insight into the metabolic changes in tobacco and soybean plants under varying levels of dehydration.  Figure 2 provides an overview of the number of metabolites from various groups detected in both plant species in different tissues. A total of 116 and 140 metabolites were detected in tobacco leaves and roots, respectively. More metabolites were detected in soybean consisting of 241 and 207 metabolites in leaves and roots, respectively. Metabolites in the amino acid and carbohydrate groups comprised the majority of the metabolites detected in both tissues for both plants. Lipids were also predominant in both tissues in soybean but only in roots in tobacco. The dominance of carbohydrates and amino acids detected in both tissues of soybean and tobacco signifies their important role in plant metabolism. Carbohydrates are an important component in plant metabolism by providing energy in various metabolic pathways and as crude materials for synthesis of other organic compounds [28]. Amino acids on the other hand can be a source of nitrogen for plant nutrition and can delay protein degradation during water deficit conditions [28, 29].

It is interesting to note that, in tobacco, abscisic acid (ABA) was only detected in roots where it accumulated significantly (8-fold; *p* < 0.001) after 240 min of water stress. This is in contrast with soybean where ABA was detected in both tissues but was only significantly accumulated in leaves after 300 min (6-fold; *p* value = 0.04) and 500 min (8-fold; *p* value = 0.002) of water stress. This appears to be a significant difference in response between the two plant species. ABA in soybean leaves accumulated up to ~100 mg/g FW when plants were stressed for 300 min [17]. This phytohormone is well documented as the key regulator of stomatal closure in plants during water stress [30]. Its biosynthesis during water stress could be in roots or leaves as reported in tomato and tobacco [31, 32]. Although no ABA was detected in our tobacco leaves, stomatal closure occurred even at the earliest stage of water stress. Holbrook et al. [31] demonstrated that ABA-deficient tomato mutants still closed their stomata during water stress. The closure of the stomata could have been triggered by hydraulic signals since stomatal closure in plants can be triggered by chemical (ABA) or hydraulic signals [30].

**Figure 3:**
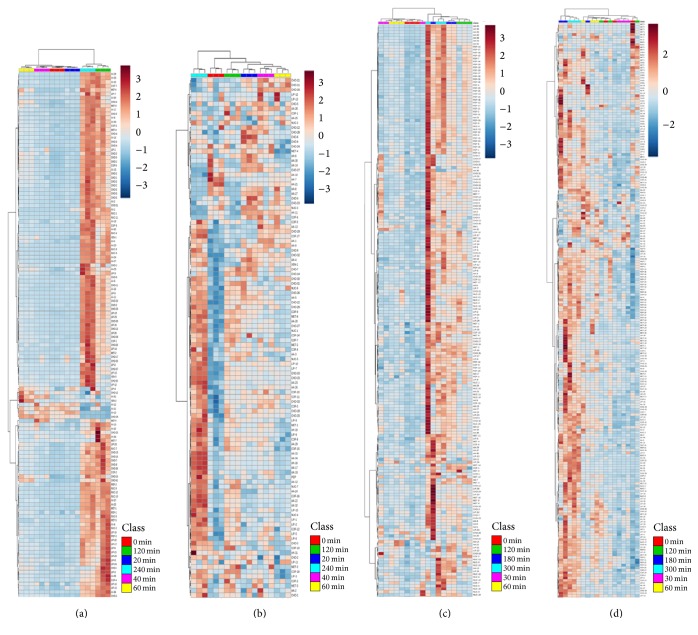
Heatmap of metabolites in tobacco and soybean leaves and roots at various dehydration time points. Tobacco roots (a) and leaves (b). Soybean roots (c) and leaves (d). In tobacco (a-b), the following colors indicate the dehydration time points: red (control), blue (20 min), pink (40 min), yellow (60 min), green (120 min), and light blue (240 min). In soybeans (c-d), the following colors indicate the dehydration time points: red (control), pink (30 min), yellow (60 min), green (120 min), blue (180 min), and light blue (300 min).

**Figure 4:**
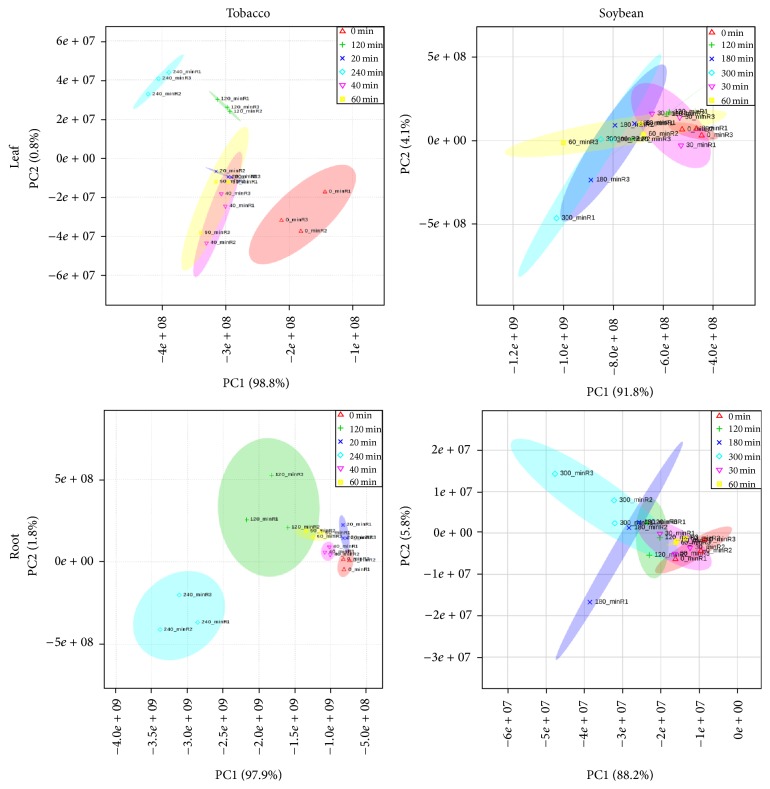
Score plots of PCA of tobacco and soybean metabolites in leaf and roots under various dehydration time points.

The general metabolome profile of the plants during the course of the progressive water stress can be seen in Figure 3. It is interesting to point out that accumulation of metabolites between two tissue samples for both species follows a different profile. Accumulation of metabolites in roots for both species was progressive (Figures 3(a) and 3(c)) and was directed towards the later stage of dehydration. Root metabolites accumulated slowly during the early stage of water stress and dramatically increased during the last two time points where the plants were experiencing extreme water stress. Hierarchical cluster analyses of root samples showed that the last two time points in tobacco (120 and 240 min) and the last three time points in soybean (120, 180, and 300 min) were distinctly separated from the rest of the time points. These trends in the dataset can be further explained by the PCA score plot (Figure 4). The scores plot of the PCA analysis showed clear groupings of the different water stress treatments. Variation in the dataset in roots in both species can be explained by principal component 1 (PC1) which accounts for 88–98% of the variation. These results showed that water stress treatments contributed significantly to the root metabolite changes. It is interesting to note that, in tobacco roots, almost all the metabolites showed progressive accumulation towards the last two time points except for nine metabolites that showed progressive accumulation up to the 60 min of dehydration and then started to decline after further dehydration. Cluster analysis clearly separated this group from the other groups based on the accumulation during the course of dehydration. These nine metabolites were composed of amino acids (asparagine, aspartate, glycine, and serine), carbohydrates (galactinol, myoinositol), lipid (1-palmitoylglycerophosphocholine), secondary metabolite (anatabine), and xenobiotic (lauryl sulfate). Looking at their accumulation in the leaf, all these metabolites are present except the xenobiotic lauryl sulfate. Galactinol and myoinositol, two precursors of raffinose, showed contrasting patterns in tobacco roots. Galactinol was not affected by water stress whereas myoinositol significantly accumulated after 40–60 min of stress and started to decline after subsequent dehydration to 240 min. Amiard et al. [33] reported a similar pattern in ryegrass under water stress. Galactinol and myoinositol accumulation were not affected by drought stress in ryegrass but instead influenced the accumulation of fructans in leaves. The four amino acids showed a downward trend in accumulation in all time points. In soybean roots, 21 metabolites have a different accumulation pattern as highlighted by cluster analysis. Most of these metabolites belong to nucleotide groups (50%). Asparagine was also clustered in this group.

Comparisons of the large metabolome datasets from tobacco and soybean show that, in contrast with the root metabolome profile, the metabolite pattern in leaves is biphasic. This pattern showed a distinct increase during the early stages of dehydration (20–40 min in tobacco; 60 min in soybean) and increased again at the late stage of dehydration. This same pattern was reflected in the profile of differentially significant metabolites as shown in Supplemental Fig. 1 in Supplementary Material available online at https://doi.org/10.1155/2017/3065251. The total number of metabolites showing a downward trend in both tissues in tobacco was minimal with numbers ranging between 0 and 9. Soybean, however, shows a contrasting trend where all the differentially significant metabolites observed showed increases. Only one compound was noted to decrease in either tissue, that is, sebacate (*p*-value = 0.0071; *q*-value = 0.3349) and genistin (*p*-value = 0.0323; *q*-value = 0.9859) in roots and leaves, respectively. Both compounds decreased significantly during the initial stage (30 min) of dehydration. Genistin, a 7-0 glycoside form of the aglycone genistein [34], is involved in the isoflavonoid pathway and significantly accumulates in the roots during the course of water stress. The same effect had been reported in soybean seeds where drought increased genistin content in seeds by 47% [35].

### 3.3. Metabolite-Metabolite Correlation Analysis

**Figure 5:**
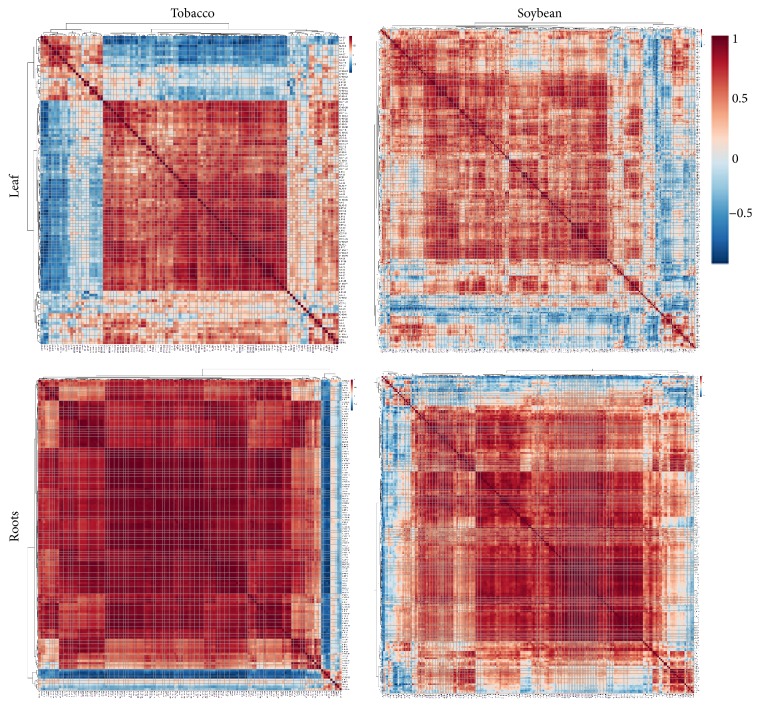
Pearson product-moment correlation analysis of tobacco and soybean metabolites measured in leaves and roots at various dehydration time points. Red color indicates positive correlation and blue indicates negative correlation.

Correlation analyses were carried out to identify the relationship among the metabolites detected in the two plant species. The heatmaps of the correlation analysis provide an overview of the correlation in each tissue for both species (Figure 5). In tobacco roots, most of the metabolites measured showed positive correlation and only nine compounds (6%) showed negative correlations and clustered in the same group. This group is the same set of metabolites detected by cluster analysis in Figure 3 to have a different accumulation pattern with the rest of the metabolites. The negative correlation of these compounds with the rest of the metabolites explained why these nine metabolites showed a distinct accumulation pattern as shown in Figure 3. The same observation was noted in soybean roots wherein 18 metabolites clustered together because they have a negative correlation with most of the metabolites detected. Thirty-nine percent of these metabolites belong to the nucleotide group. One metabolite in that cluster of 18 metabolites is asparagine. Asparagine was consistently negatively correlated with the majority of the root metabolites in both species. Its accumulation in tobacco roots positively correlates with only four metabolites, glycine, aspartate, myoinositol, and galactinol, with glycine showing the highest correlation (*r* = 0.64). In soybean, it has a positive correlation with glutamine, choline, glucose 6-phosphate, dehydroascorbate, and allantoic acid, with glutamine showing the highest correlation (*r* = 0.76). Asparagine and glutamine are the primary nitrogen transport in plants and are involved in various biological processes [36]. Although their accumulation showed a strong positive correlation, glutamine in soybean roots was not influenced by water stress while asparagine significantly accumulated during the initial stage of water stress (3-fold, *p* = 0.03) and started to decline after 60 min of dehydration. Asparagine had been reported to increase in rice during drought but its accumulation negatively correlates with water use efficiency and yield [37]. This may be a combinatorial effect from other metabolites and not solely due to the accumulation of asparagine. On the other hand, in leaves, ~23 metabolites (20% of the total metabolites detected) showed negative correlation profiles in which most of these metabolites belong to the carbohydrate and amino acid groups. A similar case was observed in soybean leaves where 39 metabolites (16% of the metabolites detected) were negatively correlated with the rest of the metabolites. Metabolites from the amino acid and carbohydrates groups were the most predominant in this group.

### 3.4. Plant and Tissue-Specific Metabolites

**Figure 6:**
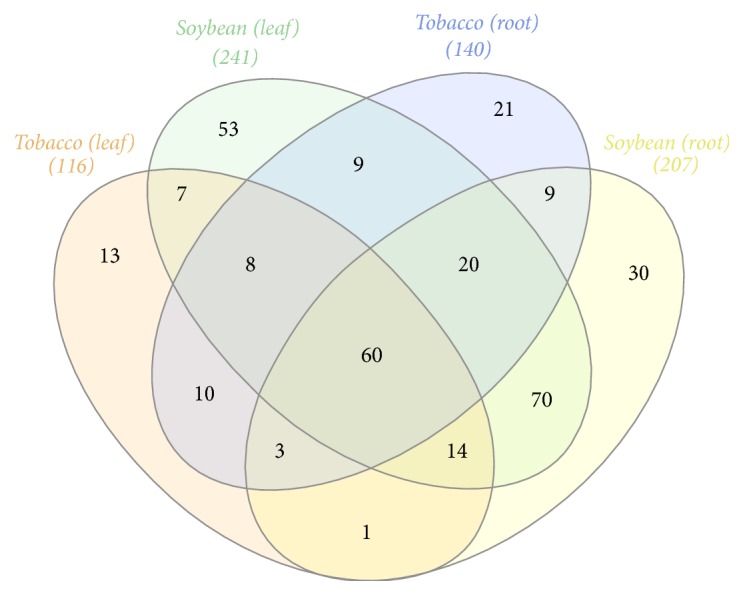
A Venn diagram representing the distribution of metabolites in tobacco and soybean leaves and root samples. Numbers in parenthesis are the total number of metabolites detected in each sample.

One of the objectives of our study is to identify potential biomarkers that can be used for screening stress response in plants. To achieve this objective, we analyzed the metabolites for their tissue-specific pattern in both species.  Figure 6 provides an overview of the number of metabolites specific and common between tissues and species. A total of 60 metabolites were conserved and shared by both plants in both tissues. Most of these metabolites belong to amino acid (33%) and carbohydrates (40%) groups. Some of these metabolites conserved in both species include myoinositol, gamma-aminobutyrate (GABA), proline, mannitol, sucrose, and pyruvate (Table 1). Soybean tissues showed more tissue-specific metabolites than tobacco. Fifty-three metabolites were found specific to soybean leaves which comprise mostly metabolites belonging to the amino acid and secondary metabolism groups (Supplemental Fig. 2). Some caution should be exercised, however, because not being detected in the tissue might not always indicate that the metabolite is not present and could just be the case that the metabolite level is below the detection limit. For example, arabinose was only detected in tobacco leaves in our study but has been reported to be abundant in vacuoles in soybean leaves [38]. It has also been reported to increase in coffee leaves when plants were under heat stress [39]. This is certainly not the case for the arabinose detected in our tobacco leaves since its accumulation was not affected by water stress. Sorbitol, which was only detected in soybean leaves, had been reported to facilitate osmotic adjustment in fruit trees [40].

In addition to qualitative differences in metabolic responses, there are also differences in the kinetics of response. For example, another osmoprotectant that was only detected in roots in both species is trehalose. It only accumulated significantly (19-fold; *p* = 0.0127) at a late time point after tobacco plants were under stress for 240 min. In soybean roots, its accumulation was earlier and significantly increased starting at 120 min of dehydration. Trehalose is a well-studied metabolite associated with abiotic stress and the subject of various metabolic engineering efforts to enhance drought tolerance in plants. Overexpression of genes involved in trehalose metabolism increased drought tolerance in rice [41] and tomato [42].

One objective of nontargeted metabolome profiling is to identify biomarkers that can be used in screening germplasm materials for tolerance to stress. In our study, we were able to identify species-specific metabolites that significantly accumulated in the roots and have the potential to be used as biomarkers for screening drought responses. In tobacco, 4-hydroxy-2-oxoglutaric acid (KHG) accumulated as high as 70-fold after 240 min of dehydration. This metabolite may therefore be a marker to differentiate sensitive from tolerant varieties subjected to water stress. There are no reports of a role in plants for KHG during water stress and this suggests that further detailed studies on the role of KHG are warranted. In soybean, coumestrol accumulated up to 161-fold higher than control levels after 180 min of dehydration. Its accumulation is 46-fold after just 60 min of dehydration. This level of accumulation, especially after only a short duration of water stress, could be a good early indicator of drought in soybean roots. Other potential biomarkers could be known stress metabolites like gamma-aminobutyric acid (GABA). GABA is a well-reported nonproteinogenic amino acid that responds to biotic and abiotic stress. Most of the metabolites involved in the GABA shunt pathway in both species were induced by water stress (Supplemental Fig. 3). The role of GABA during abiotic stress is not well understood and researchers have tried to elucidate the physiological mechanism involved by exogenous application of GABA to mitigate the effect of drought. Krishnan et al. [43] reported that exogenous application of GABA mitigates drought damage in perennial ryegrass by maintaining higher relative water content and stable membranes.

## 4. Conclusion

Two metabolomics datasets presented in this study were generated by growing both plant species in similar hydroponic systems and water deficit conditions and analyzed using identical metabolomics pipeline. Comparisons between the two datasets therefore reveal common responses between the two species, responses specific to one of the species, responses that occur in both root and leaf tissues, and responses that are specific to one organ. Some of the metabolites that increase during drought in both species include myoinositol, gamma-aminobutyrate (GABA), proline, mannitol, sucrose, and pyruvate (Table 1). Among the metabolites that show increases that are restricted to just one plant, 4-hydroxy-2-oxoglutaric acid in tobacco roots (increased 70-fold after 240 min of dehydration) and coumestrol in soybean roots (161-fold higher after 180 min) show high level tissue-specific accumulation that suggests that these two compounds could serve as biomarkers for drought stress in these two economically important species. We have previously reported the metabolomics result from each of these two species. However, the comparisons of these two large nontargeted metabolomics datasets provide novel information and detailed comparative overview at species and tissue level. Also, these comparative findings suggest that KHG will be a useful marker for drought stress for some members of Solanaceae and coumestrol for some legume species. Tobacco is a well-studied model plant for Solanaceae family and also an important economic crop like soybean. Global profiling and comparison at the metabolite level will be a useful resource for the plant community and research groups working with metabolites to have drought tolerant crops.

## Supplementary Material

Supplemental Figure 1. Total number of metabolites with significant fold change in both tissues in tobacco and soybean. Supplemental Figure 2. Percentage distribution of different groups of metabolites observed on each sample tissue on each crop. Supplemental Figure 3. GABA Shunt Pathway showing the accumulation of different metabolites involved in the pathway during the course of dehydration in both plants.

## Figures and Tables

**Table 1:** List of metabolites common to each plant and tissue samples.

Species	Tissue	Number of metabolites	Metabolites
Tobacco	Leaf	13	N-Acetylputrescine, N-delta-acetylornithine^*∗*^, pyroglutamine^*∗*^, 1,6-anhydroglucose, arabinose, nicotianamine, thiamin (vitamin B1), 9-HODE, fucosterol, heptanoate (7 : 0), cycloartenol, tryptamine, Trizma acetate
	Root	21	1,2-Propanediol, 1-stearoylglycerol (1-monostearin), 2-palmitoylglycerophosphoethanolamine^*∗*^, 3-hydroxypyridine, 3-methoxytyramine, 9,10-epoxystearate, benzyl alcohol, dihomolinoleate (20 : 2n6), dimethylglycine, gentiobiose, glucoheptose, glucosamine, glutathione, oxidized GSSG, lactate, lauryl sulfate, mannosamine, N-6-trimethyllysine, rhamnose, sedoheptulose-7-phosphate, triethanolamine, xylulose

Soybean	Leaf	53	1-Palmitoylglycerophosphate, 12,13-hydroxyoctadec-9(Z)-enoate, 2-aminobutyrate, 4-acetamidobutanoate, afromosin, alpha-amyrin, apigenin, benzene-1,3,5-triol, biochanin A, citraconate, cyanoalanine, erlose, erythrose, ferulate, galactitol (dulcitol), galactonate, gamma-glutamylalanine, gentisate, glycerophosphorylcholine (GPC), homoserine, homoserine lactone, isocitrate, isoleucylglycine, isorhamnetin, kaempferol, kaempferol 3-O-beta-glucoside, kaempferol 7-O-glucoside, kynurenate, luteolin, lysylleucine, maltotriose, methyl-beta-glucopyranoside, N-acetylglutamate, N-acetylproline, N-acetylserine, naringenin-7-O-glucoside, O-acetylserine, orotate, pheophorbide A, pyridoxate, pyroglutamylvaline, quercetin, quercetin-3-galactoside, quercetin-3-o-glucoside, rutin, S-adenosylhomocysteine (SAH), salicylate, sorbitol, squalene, *trans*-4-hydroxyproline, urate, vanillin, xylitol
	Root	30	13-Methylmyristic acid, 2,4-dihydroxybenzoic acid, 2-aminoadipate, 4-hydroxybenzyl alcohol, adenosine 3′-monophosphate (3′-AMP), allantoic acid, behenate (22 : 0), caproate (6 : 0), carboxyethyl-GABA, choline, flavin adenine dinucleotide (FAD), gamma-glutamylisoleucine^*∗*^, gamma-glutamylleucine, gamma-glutamylphenylalanine, glutarate (pentanedioate), glycerol 2-phosphate, guanosine 3′-monophosphate (3′-GMP), hypoxanthine, isoliquiritigenin, leucylasparagine, lignocerate (24 : 0), liquiritigenin, naringenin, p-hydroxybenzaldehyde, sitostanol, threonylvaline, valylalanine, valylserine, valylvaline, xanthine

Tobacco	Leaf and root	10	Spermidine, anatabine, nicotine, 1-pentylamine, quinate, phenethylamine (isobar with 1-phenylethanamine), mannose-6-phosphate, putrescine, ribulose, *trans*-urocanate

Soybean	Leaf and root	70	Glycylleucine, alanylvaline, isoleucylleucine, galactose, pipecolate, flavin mononucleotide (FMN), isoleucylalanine, arginylphenylalanine, 4-hydroxycinnamate, chiro-inositol, valylphenylalanine, lupeol, uridine-2′,3′-cyclic monophosphate, isoleucylserine, maltose, coumestrol, nicotinamide, N-acetylasparagine, leucylisoleucine, serylphenylalanine, maltol, malonate (propanedioate), arginylleucine, sebacate (decanedioate), daidzein, leucylphenylalanine, fucose, cytosine-2′,3′-cyclic monophosphate, 2′-deoxyadenosine, 2-isopropylmalate, histidylleucine, uracil, ethylmalonate, threonylleucine, isoleucylvaline, leucylglycine, benzoate, valylleucine, valylglutamate, 9,10-hydroxyoctadec-12(Z)-enoic acid, aspartylleucine, alanylisoleucine, guanosine-2′,3′-cyclic monophosphate, formononetin, genistein, serylisoleucine^*∗*^, alanylphenylalanine, glycylisoleucine, 4-octenedioate glucarate (saccharate), 13S-hydroperoxy-9Z,11E,15Z-octadecatrienoate, 1-stearoylglycerophosphocholine, leucylglutamate, 2-hydroxystearate, 8-hydroxyoctanoate, acetoacetate, leucylserine, galactarate (mucic acid), valylisoleucine, adenosine-2′,3′-cyclic monophosphate, isoleucylisoleucine, nicotinamide, riboside^*∗*^, threonylphenylalanine, choline, phosphate, N-acetylornithine, genistin, pinitol, Isobar: 1-kestose, levan, glycylvaline, daidzin

## References

[1] Cominelli E., Conti L., Tonelli C., Galbiati M. (2013). Challenges and perspectives to improve crop drought and salinity tolerance. *New Biotechnology*.

[2] Cramer G. R., Urano K., Delrot S., Pezzotti M., Shinozaki K. (2011). Effects of abiotic stress on plants: a systems biology perspective. *BMC Plant Biology*.

[3] Rabara R. C., Tripathi P., Rushton P. J. (2014). The potential of transcription factor-based genetic engineering in improving crop tolerance to drought. *OMICS A Journal of Integrative Biology*.

[4] USDA-ERS (2012). *U.S. Drought 2012: Farm and Food Impacts*.

[5] Kang Y., Khan S., Ma X. (2009). Climate change impacts on crop yield, crop water productivity and food security—a review. *Progress in Natural Science*.

[6] Obata T., Fernie A. R. (2012). The use of metabolomics to dissect plant responses to abiotic stresses. *Cellular and Molecular Life Sciences*.

[7] Davies H. V., Shepherd L. V. T., Stewart D., Frank T., Röhlig R. M., Engel K.-H. (2010). Metabolome variability in crop plant species—when, where, how much and so what?. *Regulatory Toxicology and Pharmacology*.

[8] Saito K., Matsuda F. (2010). Metabolomics for functional genomics, systems biology, and biotechnology. *Annual Review of Plant Biology*.

[9] Fiehn O. (2002). Metabolomics—the link between genotypes and phenotypes. *Plant Molecular Biology*.

[10] Bino R. J., Hall R. D., Fiehn O. (2004). Potential of metabolomics as a functional genomics tool. *Trends in Plant Science*.

[11] Shulaev V., Cortes D., Miller G., Mittler R. (2008). Metabolomics for plant stress response. *Physiologia Plantarum*.

[12] Last R. L., Jones A. D., Shachar-Hill Y. (2007). Towards the plant metabolome and beyond. *Nature Reviews Molecular Cell Biology*.

[13] Warren C. R., Aranda I., Cano F. J. (2012). Metabolomics demonstrates divergent responses of two *Eucalyptus* species to water stress. *Metabolomics*.

[14] Krasensky J., Jonak C. (2012). Drought, salt, and temperature stress-induced metabolic rearrangements and regulatory networks. *Journal of Experimental Botany*.

[15] Verslues P. E., Sharma S. (2010). Proline metabolism and its implications for plant-environment interaction. *Arabidopsis Book*.

[16] Rabara R. C., Tripathi P., Reese R. N. (2015). Tobacco drought stress responses reveal new targets for Solanaceae crop improvement. *BMC Genomics*.

[17] Tripathi P., Rabara R. C., Reese R. N. (2016). A toolbox of genes, proteins, metabolites and promoters for improving drought tolerance in soybean includes the metabolite coumestrol and stomatal development genes. *BMC Genomics*.

[18] Oliver M. J., Guo L., Alexander D. C., Ryals J. A., Wone B. W. M., Cushman J. C. (2011). A sister group contrast using untargeted global metabolomic analysis delineates the biochemical regulation underlying desiccation tolerance in *Sporobolus stapfianus*. *Plant Cell*.

[19] Chen L. M., Zhou X. A., Li W. B. (2013). Genome-wide transcriptional analysis of two soybean genotypes under dehydration and rehydration conditions. *BMC Genomics*.

[20] Rabara R. C., Tripathi P., Choudhary M. K., Timko M. P., Shen Q. J., Rushton P. J. (2015). Transcriptome profiling of tobacco under water deficit conditions. *Genomics Data*.

[21] Tripathi P., Rabara R. C., Shen Q. J., Rushton P. J. (2015). Transcriptomics analyses of soybean leaf and root samples during water-deficit. *Genomics Data*.

[22] Wan J., Griffiths R., Ying J., McCourt P., Huang Y. (2009). Development of drought-tolerant canola (*Brassica napus* L.) through genetic modulation of ABA-mediated stomatal responses. *Crop Science*.

[23] Heschel M. S., Riginos C. (2005). Mechanisms of selection for drought stress tolerance and avoidance in *Impatiens capensis* (Balsaminaceae). *American Journal of Botany*.

[24] Rahnama A., James R. A., Poustini K., Munns R. (2010). Stomatal conductance as a screen for osmotic stress tolerance in durum wheat growing in saline soil. *Functional Plant Biology*.

[25] Hopper D. W., Ghan R., Cramer G. R. (2014). A rapid dehydration leaf assay reveals stomatal response differences in grapevine genotypes. *Horticulture Research*.

[26] Hepworth C., Doheny-Adams T., Hunt L., Cameron D. D., Gray J. E. (2015). Manipulating stomatal density enhances drought tolerance without deleterious effect on nutrient uptake. *New Phytologist*.

[27] Silveira J. A. G., Araújo S. A. M., Lima J. P. M. S., Viégas R. A. (2009). Roots and leaves display contrasting osmotic adjustment mechanisms in response to NaCl-salinity in *Atriplex nummularia*. *Environmental and Experimental Botany*.

[28] Zhao Y., Zhao C., Lu X. (2013). Investigation of the relationship between the metabolic profile of tobacco leaves in different planting regions and climate factors using a pseudotargeted method based on gas chromatography/mass spectrometry. *Journal of Proteome Research*.

[29] Bowne J. B., Erwin T. A., Juttner J. (2012). Drought responses of leaf tissues from wheat cultivars of differing drought tolerance at the metabolite level. *Molecular Plant*.

[30] Schachtman D. P., Goodger J. Q. D. (2008). Chemical root to shoot signaling under drought. *Trends in Plant Science*.

[31] Holbrook N. M., Shashidhar V. R., James R. A., Munns R. (2002). Stomatal control in tomato with ABA-deficient roots: response of grafted plants to soil drying. *Journal of Experimental Botany*.

[32] Borel C., Frey A., Marion-Poll A., Tardieu F., Simonneau T. (2001). Does engineering abscisic acid biosynthesis in Nicotiana plumbaginifolia modify stomatal response to drought?. *Plant, Cell & Environment*.

[33] Amiard V., Morvan-Bertrand A., Billard J.-P., Huault C., Keller F., Prud'homme M.-P. (2003). Fructans, but not the sucrosyl-galactosides, raffinose and loliose, are affected by drought stress in perennial ryegrass. *Plant Physiology*.

[34] Dhaubhadel S. (2011). *Regulation of Isoflavonoid Biosynthesis in Soybean Seeds*.

[35] Caldwell C. R., Britz S. J., Mirecki R. M. (2005). Effect of temperature, elevated carbon dioxide, and drought during seed development on the isoflavone content of dwarf soybean [*Glycine max* (L.) Merrill] grown in controlled environments. *Journal of Agricultural and Food Chemistry*.

[36] Lea P. J., Sodek L., Parry M. A. J., Shewry P. R., Halford N. G. (2007). Asparagine in plants. *Annals of Applied Biology*.

[37] Degenkolbe T., Do P. T., Kopka J., Zuther E., Hincha D. K., Köhl K. I. (2013). Identification of drought tolerance markers in a diverse population of rice cultivars by expression and metabolite profiling. *PLoS ONE*.

[38] Benkeblia N., Shinano T., Osaki M. (2007). Metabolite profiling and assessment of metabolome compartmentation of soybean leaves using non-aqueous fractionation and GC-MS analysis. *Metabolomics*.

[39] Lima R. B., Dos Santos T. B., Vieira L. G. E. (2013). Heat stress causes alterations in the cell-wall polymers and anatomy of coffee leaves (*Coffea arabica* L.). *Carbohydrate Polymers*.

[40] Lo Bianco R., Rieger M., Sung S.-J. S. (2000). Effect of drought on sorbitol and sucrose metabolism in sinks and sources of peach. *Physiologia Plantarum*.

[41] Redillas M. C. F. R., Park S.-H., Lee J. W. (2012). Accumulation of trehalose increases soluble sugar contents in rice plants conferring tolerance to drought and salt stress. *Plant Biotechnology Reports*.

[42] Cortina C., Culiáñez-Macià F. A. (2005). Tomato abiotic stress enhanced tolerance by trehalose biosynthesis. *Plant Science*.

[43] Krishnan S., Laskowski K., Shukla V., Merewitz E. B. (2013). Mitigation of drought stress damage by exogenous application of a non-protein amino acid *γ*-aminobutyric acid on perennial ryegrass. *Journal of the American Society for Horticultural Science*.

