# Supplementary material for: Comparative Metabolome Profile between Tobacco and Soybean Grown under Water-Stressed Conditions
Source: Biomed Res Int. 2017 Jan 3;2017:3065251. doi: 10.1155/2017/3065251 (PMC5239840; doi:10.1155/2017/3065251)
Supplement: Supplementary file 1 — Supplemental Figure 1. Total number of metabolites with significant fold change in both tissues in tobacco and soybean. Supplemental Figure 2. Percentage distribution of different groups of metabolites observed on each sample tissue on each crop. Supplemental Figure 3. GABA Shunt Pathway showing the accumulation of different metabolites involved in the pathway during the course of dehydration in both plants. [file 3065251.f1.pdf]

Supplemental Data

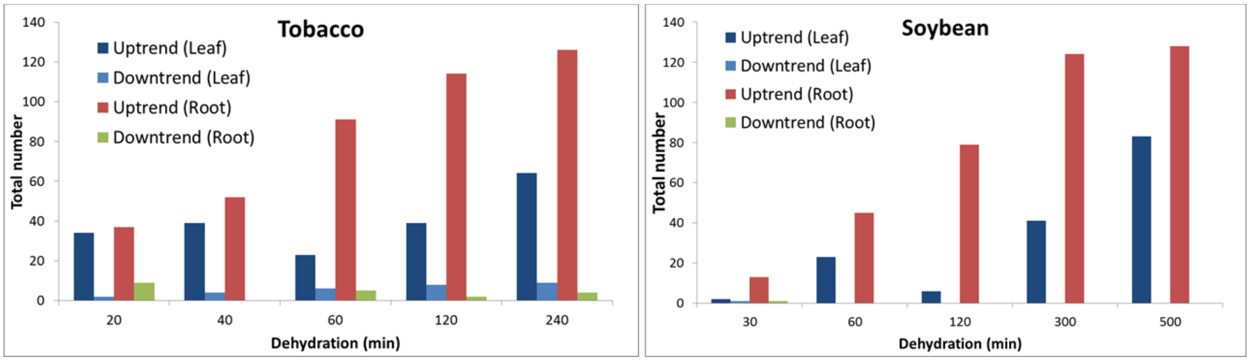

Supplemental Fig. 1. Total number of metabolites observed in tobacco and soybean leaves and roots with significant fold change (FC) that exhibit up or down trend at each dehydration time point. Significantly difference was inferred at p≤0.05using Welch's t-test.

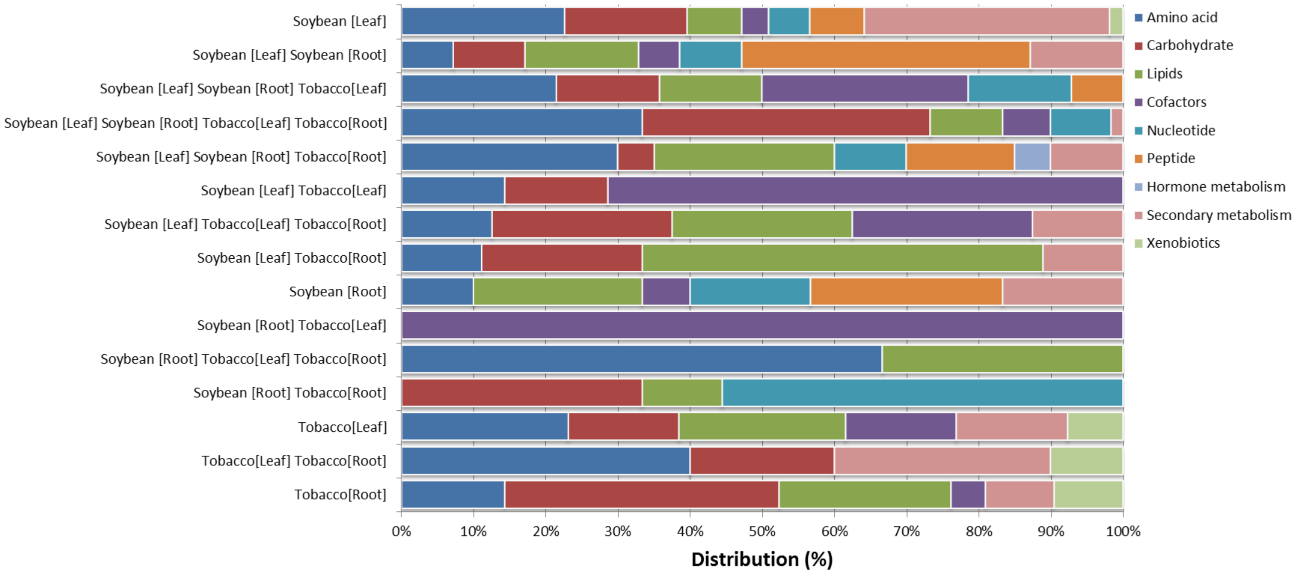

Supplemental Fig. 2. Distribution of metabolites shared between tobacco and soybean across tissue samples.

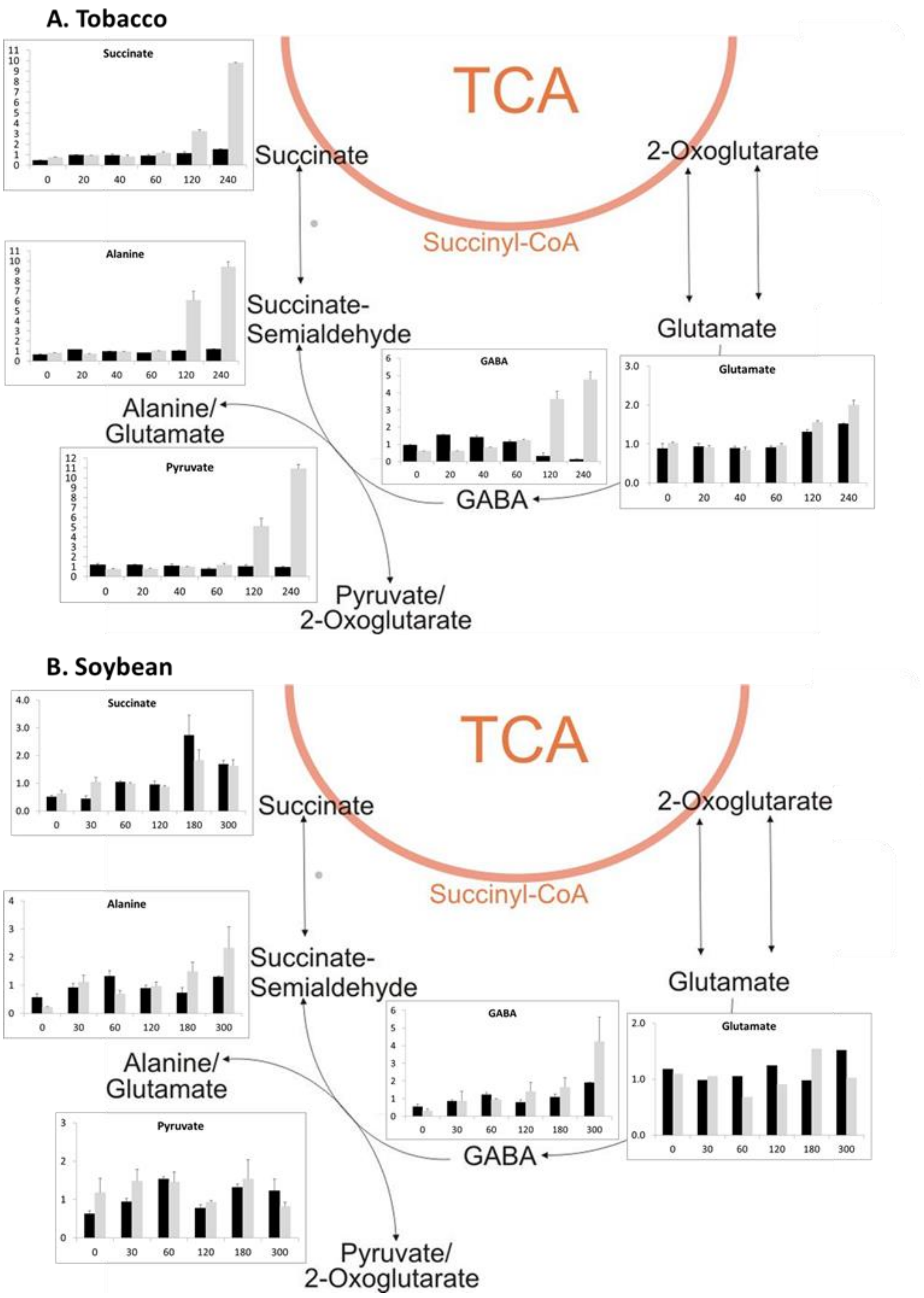

Supplemental Fig. 2. GABA shunt pathway. Leaf samples are in black and root samples are in grey. Data are mean and standard error of three biological replicates.
